# Origin of Circulating Free DNA in Sepsis: Analysis of the CLP Mouse Model

**DOI:** 10.1155/2015/614518

**Published:** 2015-07-26

**Authors:** Shigeto Hamaguchi, Yukihiro Akeda, Norihisa Yamamoto, Masafumi Seki, Kouji Yamamoto, Kazunori Oishi, Kazunori Tomono

**Affiliations:** ^1^Division of Infection Control and Prevention, Osaka University Graduate School of Medicine, Osaka 565-0871, Japan; ^2^International Research Center for Infectious Diseases, Research Institute for Microbial Diseases, Osaka University, Osaka 565-0871, Japan; ^3^Department of Clinical Epidemiology and Biostatistics, Osaka University Graduate School of Medicine, Osaka 565-0871, Japan; ^4^Infectious Disease Surveillance Center, National Institute of Infectious Diseases, Tokyo 162-8640, Japan

## Abstract

Recently, it has been reported that circulating free DNA (cf-DNA) in the blood is increased in various infectious diseases, including sepsis. Moreover, a relationship between cf-DNA and neutrophil extracellular traps (NETs) has been suggested. However, it is still unclear what the source and physiological role of cf-DNA in sepsis are. In this study, we examined the source of cf-DNA by detecting citrullinated histone H3, a characteristic feature of NET formation, in cecal ligation and puncture- (CLP-)operated mice. In addition, neutrophil depletion using anti-Ly6G antibodies was performed to assess the association between neutrophils and cf-DNA. Increased cf-DNA levels were observed only in CLP mice and not in the control groups; the qPCR findings revealed that the cf-DNA was mainly host-derived, even in bacteremic conditions. Citrullinated histone H3 was not increased in the neutrophils upon CLP, and the depletion of neutrophils showed limited effects on decreasing the amount of cf-DNA. Taken together, these results suggested that elevated cf-DNA levels during early-phase sepsis may represent a candidate biomarker for the severity of sepsis and that, contrary to previous findings, cf-DNA is not derived from neutrophils or NETs.

## 1. Introduction

Sepsis is an emergency condition associated with significant mortality and excessive inflammation [1–5]. Sepsis caused by bacteremia arises from the host response to infection. Currently, the diagnosis of sepsis caused by bacteremia relies on culture-based pathogen detection and physiological criteria, including changes in the body temperature and heart/respiration rates. While these clinical diagnostic criteria are simple and clear, novel sepsis biomarkers that can lead to a more reliable early diagnosis and therapeutic decision-making are urgently needed. Until now, a number of molecules have been proposed as candidate sepsis biomarkers; however, there are currently few useful predictive biomarkers for the severity and prognosis of sepsis available in clinical practice [6].

Recently, it was reported that the circulating free DNA (cf-DNA) levels in the blood are increased in various infectious diseases, including sepsis [7, 8]. Accordingly, cf-DNA has been suggested as a potential predictive biomarker for several different conditions, including cancer and injury [9, 10]; moreover, one study of sepsis patients reported that greatly elevated plasma DNA and nucleosome levels (>800 ng/mL) were associated with a poorer outcome [7].

Furthermore, recent studies have reported that cf-DNA is associated with neutrophil extracellular traps (NETs) [7, 8, 10]. NETs were first reported as a novel innate immune mechanism of neutrophils in 2004 [11]. These are fibrous mesh-like structures that can rapidly trap and kill microbial pathogens [12]. In activated neutrophils, a mixture of chromosomal DNA and intracellular contents is extruded to the extracellular space as a fibrous structure upon a variety of proinflammatory stimuli [13–15]. Moreover, citrullinated histone H3 has been reported as a characteristic molecule involved in NET formation* in vitro*, with citrullination of histone H3 by peptidylarginine deiminase 4 playing a pivotal role in chromatin decondensation during “NETosis” [15, 16]. In terms of the contribution of NETs to the host defense, it has been reported that depletion of NETs can lead to hypersusceptibility to polymicrobial sepsis in mice [17]. Taken together, these previous findings suggest a potential relationship between cf-DNA and NETs in various aspects. However, it should be noted that most of the previous studies on cf-DNA were conducted using only human samples, and whether cf-DNA is indeed derived from NETs remains unclear.

In this study, using a mouse cecal ligation and puncture (CLP) sepsis model, we confirmed the elevation of the cf-DNA levels during sepsis and investigated whether the source of this cf-DNA was neutrophils, with particular focus on the NETs, or not.

## 2. Materials and Methods

### 2.1. Cecal Ligation and Puncture (CLP) Mouse Model

The CLP model for polymicrobial sepsis developed by Chaudry et al. [18] was established as previously described [19], with some modifications. In brief, 7-8-week-old C57BL/6J mice were used for the CLP operation; these were housed under specific pathogen-free conditions with free access to standard rodent food and water. We used five C57BL/6J mice per group for each experiment, except for the experiment shown in Figure 2(a), for which 13 mice in each group were used. Under general anesthesia, midline laparotomy was performed and the cecum was exposed and ligated distal to the ileocecal valve to prevent bowel obstruction, and the distal part of the cecum was punctured with a 22-gauge needle. A small amount of cecal content was manually extruded from the punctured cecum into the abdominal cavity. In all studies, after returning the cecum into the abdomen, 1 mL of phosphate-buffered saline (PBS) for fluid resuscitation was administered to create a more clinically relevant sepsis model as the standard care for human operations. The abdomen was closed using a single-layer technique. The sham mice were treated identically as the operated mice with the exception of the ligation and puncture of the gut. Under these conditions, all CLP mice showed signs of severe illness within 24 hours after the operation and high lethality after 48 hours. Further, treatment-naïve mice were included as additional controls for some experiments. All animal experiments were conducted in accordance with the Institutional Animal Care and Use Committee Guidelines of Osaka University.

### 2.2. Measurement of Plasma cf-DNA Amount

Whole blood was collected from each mouse by cardiac puncture under anesthesia and transferred into ethylenediaminetetraacetic acid-2Na tubes. The plasma was separated by centrifugation at 800 g for 10 minutes and immediately frozen at −80°C.

In order to explore the dynamics of cf-DNA under septic conditions, plasma was collected at 6 and 24 hours after the CLP operation from all mice, and the amount of cf-DNA in the plasma at each time point was quantified directly using the Quant-iT PicoGreen dsDNA Quantification Reagent Kit (Molecular Probes, Leiden, The Netherlands) and a fluorescence microplate reader (SH-9000 Lab, Hitachi High-Technologies, Tokyo, Japan) according to the manufacturers' instructions. PicoGreen specifically binds dsDNA, and after excitation at 485 nm, the dsDNA/PicoGreen fluorescence complex can be detected at 538 nm [20].

### 2.3. Measurement of Plasma Interleukin-6 (IL-6)

The plasma IL-6 levels were measured by enzyme-linked immunosorbent assay (Quantikine mouse IL-6 immunoassay kit; R&D Systems, Wiesbaden, Germany) according to the manufacturer's instructions.

### 2.4. Counting the Numbers of Bacteria

Bacteremia in the CLP mouse model was confirmed through analysis of whole blood immediately obtained by cardiac puncture. The local bacterial load in the CLP mice was determined by 10-fold serial dilution to a maximum of 10^8^. All samples were plated on sheep blood agar plates and incubated overnight at 37°C under aerobic conditions. The numbers of bacteria were determined by manual counting of the colonies on the plates.

### 2.5. Measurement of Bacterial/Host-Derived DNA

DNA was purified from the mouse plasma using the QIAamp Blood DNA Midi Kit (Qiagen, Venlo, The Netherlands) in accordance with the manufacturer's instructions. To quantify the estimated amount of DNA in the plasma, 16S rDNA and mouse *β*-2-microglobulin (B2M) were selected as representative targets of bacterial DNA and mouse-derived DNA, respectively, and TaqMan quantitative polymerase chain reaction (qPCR) assay of the purified DNA was subsequently performed using an ABI 7900HT real-time PCR machine (Applied Biosystems, Foster City, CA, USA). Amplification of each target was performed using the KAPA SYBR fast qPCR kit (Kapa Biosystems, Inc., Woburn, MA, USA) with the following primers: 16S-forward primer (1055f: 5′-ATGGCTGTCGTCAGCT-3′), 16S-reverse primer (1392r: 5′-ACGGGCGGTGTGTAC-3′), B2M-forward primer (B2M exon4-F; 5′-CTTTTGGTAAAGCAAAGAGGCC-3′), and B2M-reverse primer (B2M exon4-R; 5′-TTGGGGGTGAGAATTGCTAAG-3′) [21]. The reaction mixture (8 *μ*L) contained 5 *μ*L of KAPA SYBR qPCR Master Mix, 0.2 *μ*L of distilled water, 0.4 *μ*L of each 5 *μ*M primer, and 2 *μ*L of sample DNA. PCR was performed at 95°C for 60 seconds, followed by 40 cycles of 95°C for 15 seconds, 60°C for 60 seconds, and final extension at 72°C for 15 seconds. The results were analyzed using SDS 2.3 software (Applied Biosystems). A standard curve was determined based on the concentration gradient of the purified DNA from mouse whole blood for mice B2M and the purified DNA from* Streptococcus pneumoniae* for 16S rDNA.

### 2.6. Blood Cell Counting and Isolation of Neutrophils from Mice

For each mouse, 20 *μ*L of blood was analyzed to count the number of white blood cells (WBCs) using the Celltac hematology analyzer (MEK-6308; Nihon Kohden, Tokyo, Japan).

For neutrophil isolation, blood was collected from each mouse followed by separation with centrifugation for 20 minutes at 800 g using Histopaque-1119 (Sigma-Aldrich, St. Louis, MO, USA). The neutrophil-rich phase was collected, washed in PBS, and separated by discontinuous density-gradient centrifugation in Percoll (GE Healthcare, Buckinghamshire, UK), as previously described [22]. Subsequently, the neutrophils were collected from the 70–75% layer of the Percoll gradient and washed with PBS; after hypotonic lysis with 0.2% and 1.6% NaCl solutions to remove residual erythrocytes, the cells were resuspended in RPMI-1640 (Invitrogen, Waltham, MA, USA). Purity and viability were routinely assessed using Diff-Quik stain (Sysmex, Kobe, Japan) and trypan blue stain (Wako Pure Chemical Industries, Osaka, Japan), respectively, under a microscope.

### 2.7. Detection of Citrullinated Histone H3 as a NET Marker

For flow cytometric analyses, isolated neutrophils were fixed in 2% paraformaldehyde for 5 minutes and washed three times in PBS with 3% fetal bovine serum (FBS). Intracellular citrullinated histone H3 staining was carried out as follows: fixed neutrophils were incubated (10^6^ cells/mL) in RPMI-1640 (Sigma-Aldrich) with 1% FBS for 30 minutes in the presence of 0.5% saponin. After washing with PBS, the cells were incubated with Alexa488-conjugated rabbit citrullinated histone H3 antibody (ab5103; Abcam, Cambridge, UK) using the Alexa Fluor 488 Monoclonal Antibody Labeling Kit (Invitrogen) or isotype control antibody (sc-45068; Santa Cruz Biotechnology Inc., Santa Cruz, CA, USA) for 20 minutes and then washed in PBS with 3% FBS. The cells were analyzed using fluorescence-activated cell sorting (FACS) Calibur with CellQuest software (BD Biosciences, San Jose, CA, USA).

For western blotting, cell lysates of Percoll gradient-purified neutrophils were subjected to SDS-PAGE and transferred to a PVDF membrane blocked with Can Get Signal blocking buffer (TOYOBO, Osaka, Japan) and incubated with goat polyclonal anti-histone H3 antibody (ab5103, Abcam; 1 : 1000 in tris-buffered saline-Tween 20 [TBST]) or rabbit polyclonal anti-citrullinated histone H3 antibody (ab12079; Abcam; 1 : 1000 in TBST) for 1 hour at room temperature. The proteins were detected upon incubation with horseradish peroxidase-conjugated anti-goat or anti-rabbit IgG (1 : 10,000) for 15 minutes using ECL Western Blotting Reagent (GE Healthcare).

### 2.8. Neutrophil Depletion* In Vivo*

Ly6G-specific monoclonal antibody was used to specifically deplete neutrophils in the mice [23]. The mice were intraperitoneally injected with 1 mg of isotype control rat IgG or rat monoclonal anti-Ly6G antibody (1A8; BioXCell, Lebanon, NH, USA) 2 days before and on the day of CLP operation. After neutrophil depletion, the mice were operated for CLP or sham operation. Subsequently, the mice were sacrificed and the whole blood and intraperitoneal wash from each mouse were subjected to cell counting at 6 and 24 hours after the operation. Neutrophil depletion was confirmed by morphology using Diff-Quik stain and by cell counts using the Celltac hematology analyzer.

### 2.9. Statistical Analysis

**Figure 1:**
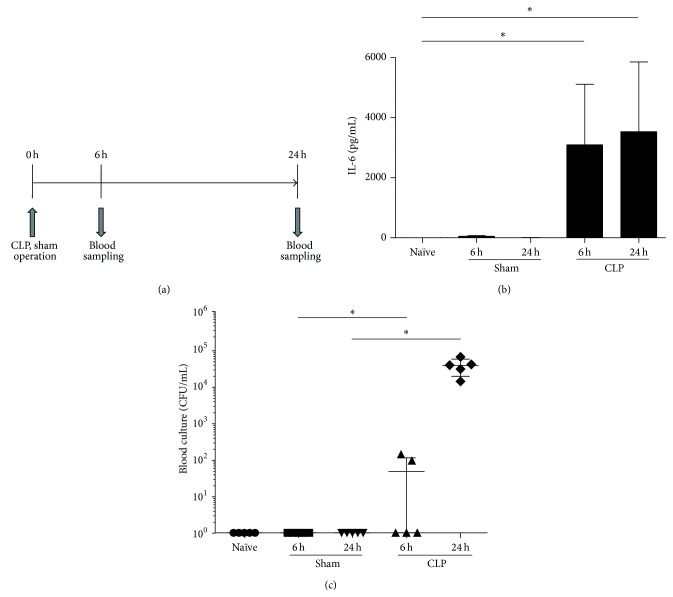
Establishment of sepsis in the cecal ligation and puncture (CLP) mice. (a) Plasma levels of interleukin-6 (IL-6) at 6 and 24 hours after CLP or sham operation. (b) Bacterial counts of the blood culture at 6 and 24 hours after CLP or sham operation. Five mice from each group were used for each experiment. The error bars represent the mean ± standard deviation; ^*∗*^ *P* < 0.05.

**Figure 2:**
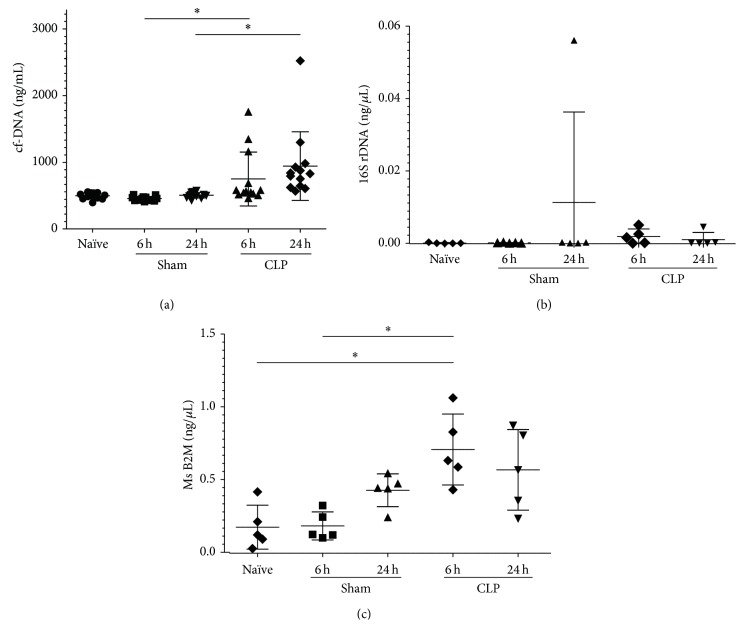
Dynamics of circulating free (cf) DNA and quantification of host-derived and bacteria-derived cf-DNA. (a) Amount of cf-DNA in the plasma at 6 and 24 hours after cecal ligation and puncture (CLP) or sham operation (*n* = 13 mice per group). (b) Amount of bacteria-derived DNA, quantified based on 16s rDNA by quantitative real-time polymerase chain reaction (PCR) (*n* = 5 mice per group). (c) Amount of host-derived DNA, quantified based on mouse beta-2-microgloblin by quantitative real-time PCR (*n* = 5 mice per group).

Statistical analyses were performed using GraphPad Prism (version 5.02; GraphPad Software, San Diego, CA, USA). All data are presented as mean ± standard deviation. Data obtained from multiple groups were tested using the nonparametric Kruskal-Wallis test followed by post hoc Dunn's multiple comparison tests. Data were considered to be statistically significant at *P* < 0.05. In the Figures 1, 2, and 4, statistical significance is indicated by asterisks.

## 3. Results

### 3.1. Establishment of Septic Condition in CLP Mice

For each experiment, CLP or sham operation was performed in five C57BL/6J mice each, and blood samples were collected at 6 and 24 hours after the operation. In the CLP mice, the plasma IL-6 levels were found to be increased 6 and 24 hours after CLP. In contrast, plasma obtained from the sham-operated mice and naïve mice contained extremely low levels of IL-6 (Figure 1(a)). Likewise, no local bacterial load was detected from any of the blood cultures from these mice. On the other hand, for the CLP mice, 2 out of 5 blood cultures were positive at 6 hours, and these contained approximately 1 × 10^2^ CFU/mL; at 24 hours, all samples (5/5) were positive for bacterial growth (approximately 1 × 10^5^ CFU/mL; Figure 1(b)). These results confirmed that CLP mice at 6 and 24 hours after CLP operation are under septic conditions [17] and can be used as a septic model for further analyses.

### 3.2. Dynamics of cf-DNA under Septic Conditions in CLP Mice

The amount of cf-DNA gradually increased at 6 hours after the CLP operation and peaked at 24 hours. In contrast, the cf-DNA levels were not elevated in the sham-operated mice and naïve mice (Figure 2(a)). Significant differences between the CLP and sham groups were observed at each time point. As the elevation of the cf-DNA amount may have been caused by bacteremia, the origin of the cf-DNA was determined by qPCR of the plasma cf-DNA using organism-specific primers (mouse B2M and bacterial 16S rDNA) [24]. The amount of 16S rDNA was slightly increased in the plasma from the CLP group at 6 hours after CLP (Figure 2(b)), whereas the amount of B2M was significantly increased in the plasma from the CLP group at 6 and 24 hours after CLP (Figure 2(c)). Similar increments in the amount of B2M were observed in each CLP group, with the amount of 16S rDNA being only approximately 1% of that of mouse B2M at each time point. These results suggested that, even under septic conditions, the cf-DNA was mainly derived from the host cells.

### 3.3. Association between Neutrophils and Serum cf-DNA Elevation under Septic Conditions

**Figure 3:**
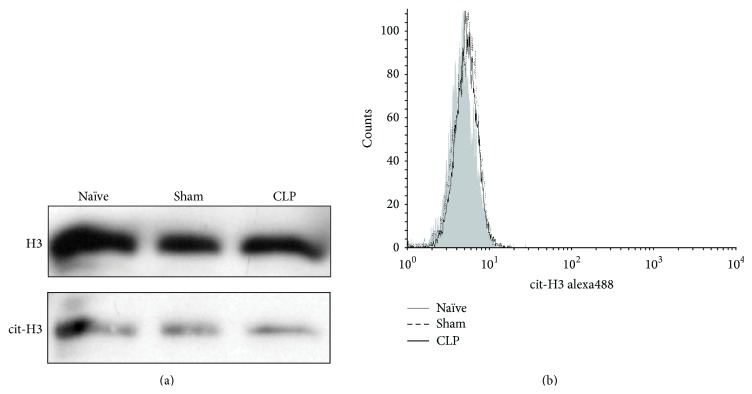
Involvement of citrullinated histone H3 in sepsis. (a) Western blot analysis of histone H3 and citrullinated histone H3 after cecal ligation and puncture (CLP) or sham operation. (b) Flow cytometric analysis of citrullinated histone H3 after CLP or sham operation. The samples were collected from a single mouse from each group, and the experiments were repeated at least three times.

It has been proposed that neutrophils contribute to the elevation of cf-DNA in the blood; however, it has not been demonstrated whether neutrophils, or more precisely NETs, correlate with increased cf-DNA levels under septic conditions. Recently, it was reported that citrullinated histone H3 represents a likely hallmark of NET formation via peptidylarginine deiminase 4. To test this hypothesis, whole blood was collected from the CLP mice, and the neutrophils were isolated and subjected to western blotting and FACS analysis using anti-citrullinated histone H3 as a NET marker. Compared with the neutrophils of naïve mice, minimal differences in terms of the size of the anti-citrullinated histone H3 antibody bands were observed upon western blotting (Figure 3(a)). Similarly, FACS analysis also revealed no shifts of the fluorescent peaks of citrullinated histone H3 between sham-operated and CLP mice as compared with naïve mice (Figure 3(b)).

### 3.4. Dynamics of cf-DNA in Neutrophil-Depleted Mice

**Figure 4:**
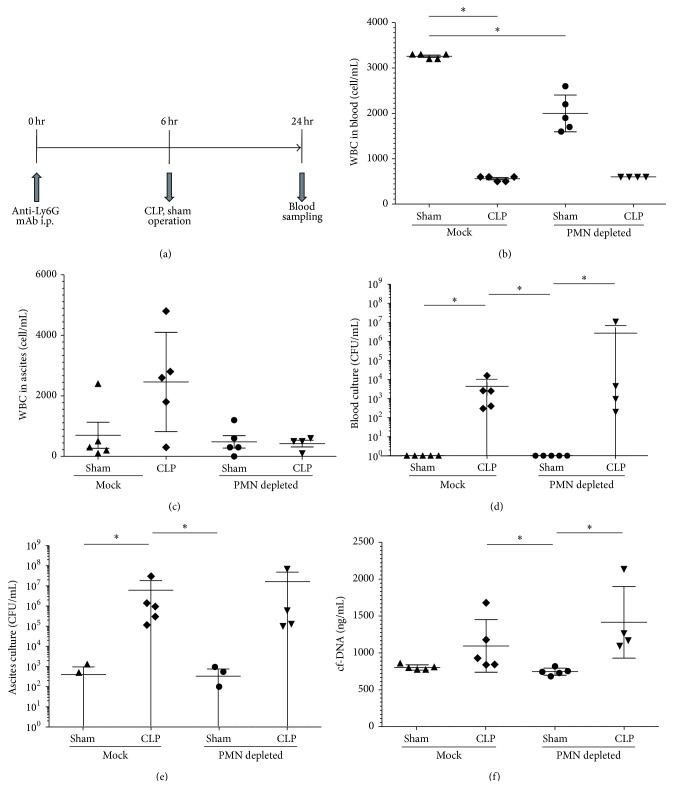
Effect of neutrophil depletion on circulating free (cf) DNA. (a), (b) White blood cell number in (a) the blood and (b) ascites at 24 hours after the operation, with or without neutrophil depletion. (c), (d) Bacterial count of (c) the blood and (d) ascites cultures at 24 hours after the operation, with or without neutrophil depletion. (e) Amount of cf-DNA in the plasma at 24 hours after cecal ligation and puncture (CLP) or sham operation, with or without neutrophil depletion. Five mice from each group were used for each experiment.

To assess the involvement of neutrophils, the neutrophils were depleted by injection of anti-Ly6G antibodies [23]. When the anti-Ly6G antibody was injected to the sham-operated group, their WBC numbers in the blood decreased, with less than 2% neutrophils observed in the WBC at 6 and 24 hours by Diff-Quik stain of the blood smear, confirming successful depletion of neutrophils. Using the same protocol of neutrophil depletion, the neutrophil-depleted CLP-operated mice showed significantly lower numbers of WBCs in the blood than those of the sham-operated mice. However, the CLP-operated group treated with the control antibody showed similar low numbers of WBCs in the blood, which may have resulted from the consumption of the WBCs in the circulation at inflammation sites under septic condition (Figure 4(a)). On the other hand, the WBC counts in the ascites from the CLP-operated group with neutrophil depletion showed lower cell counts than those of the CLP-operated group treated with the control antibody (Figure 4(b)). Along with the WBC counts, the bacterial load was also examined and indicated that neutrophil depletion had no effect on the bacterial load in the blood and ascites with versus without neutrophils (Figures 4(c) and 4(d)). Finally, the amount of cf-DNA in each group was measured and revealed that the neutrophil depletion did not affect the amount of cf-DNA in the blood under septic conditions (Figure 4(e)). Taken together, these results suggested that the increasing levels of cf-DNA in the blood under septic conditions were derived from host cells other than neutrophils, which have been reported as a likely source of cf-DNA by NET formation.

## 4. Discussion

The presence of abnormally high levels of cf-DNA in the plasma of patients with malignant diseases was first described in the 1970s [18]. However, it is only recently that cf-DNA has attracted attention in terms of its potential use as a diagnostic or prognostic marker [6, 9].

In this study,* in vivo* experiments using a CLP mouse model of sepsis showed that the cf-DNA levels increased in a time-dependent manner after the onset of sepsis (Figure 2(a)). This finding confirmed the conclusion of previous clinical studies reporting that cf-DNA correlated with the severity of the clinical outcome of sepsis [7, 8]. cf-DNA is found in many pathophysiological conditions, including infection and cancer [7, 9, 25–27]. These conditions generally involve apoptosis and/or necrosis; therefore, it is reasonable to consider apoptosis and necrosis as sources for the presence of cf-DNA [28]. To date, cf-DNA has been evaluated primarily as a biomarker of septic condition, whereas the potential function of cf-DNA has not been investigated in detail. Our results indicate that the sepsis model used in this study, the CLP mouse model, is sufficient as an evaluation model for cf-DNA.

As another important result, we found that the increase in cf-DNA in CLP mice contained mostly host cf-DNA and only negligibly amounts of bacterial DNA. This finding suggests that the cf-DNA was mainly derived from the host cells even under septic/bacteremic conditions. Further, our study investigated whether NET formation occurred during sepsis in our model, using citrullination of histone H3, a known NET marker. Interestingly, two independent experiments (western blotting and FACS analysis) showed that citrullinated histone H3 was barely increased under septic conditions. As mentioned, citrullinated histone H3 has been reported as a characteristic feature of NET formation and has been specifically implicated in the decondensation of the nucleus [16, 29]. Therefore, our results suggested that, under septic conditions, the increase of cf-DNA might not be derived from the NETs produced by neutrophils, but rather from other types of host cells. Although the relationship between citrullinated histone H3 and NET formation was recently demonstrated* in vitro*, it has been suggested that the presence of citrullinated histone H3 is not an ultimate prerequisite for the formation of NETs within individual neutrophils and the absence of an increase in the amount of citrullinated histone H3 in neutrophils observed in the present study implies a lack of involvement of NETs in cf-DNA production under* in vivo* septic conditions. Although we originally hypothesized that neutrophils were the main source of cf-DNA, as the citrullination of histone did not increase even under septic conditions, this hypothesis was refuted. Additionally, our hypothesis was also refuted by the results of the neutrophil depletion. Previously, our group observed the presence of NETs in circulating blood under systemic inflammatory response syndrome conditions, including sepsis, using fluorescent immunohistochemical analysis of blood smears [30], and several other studies have reported similar findings, hence suggesting a potential relationship between NETs and cf-DNA [7, 8, 10, 17]. Therefore, the results acquired in the present study were surprising for us, as we expected that the cf-DNA would comprise mainly NET-derived DNA. The exact mechanism of cf-DNA production in the blood is still not understood; although our experiments did not reveal the cells of cf-DNA origin, our findings do indicate that NETs do not participate in the production of cf-DNA, at least not under severe bacteremic conditions. This discrepancy in terms of the source of cf-DNA will need to be examined and confirmed by further experiments in the future.

Nonetheless, we speculate that a potential source of cf-DNA might be necrotic tissue or apoptotic cells at the infection site, or, more specifically, endothelial cells. In fact, a relationship between cf-DNA and plasma levels of typical cellular apoptotic markers has been described in lung cancer patients [31]. Moreover, an association between NET-related endothelial damage and platelets has also been described [32].

To study the innate immune mechanisms, host-derived and bacterial DNA must be distinguished. In addition to citrullinated histone H3 used in this study, other mechanisms to accurately recognize host DNA, including NETs, such as detection of specific proteins like Toll-like receptor-9 or detection of ds-DNA or the CpG motif, may prove useful in future experiments. In innate immune mechanism, it is strictly controlled to distinguish between host-derived DNA and bacterial DNA. It would be possible to find out another unknown mechanism to recognize host DNA including NETs by specific protein such as cit-H3, besides Toll-like receptor-9 to detect ds-DNA of CpG motif.

From our results and those of the previous reports on the topic, we believe that cf-DNA shows potential as a noninvasive, useful biomarker of sepsis and bacteremia. However, the significance of cf-DNA under sepsis has not yet been fully investigated. Our experiments indicated that the CLP mouse model is a promising model for studying the significance of cf-DNA. In the future, using this model along with different animal models of sepsis, the mechanism of cf-DNA production should be clarified to confirm the utility of cf-DNA as a biomarker for sepsis, and clinical research on the relationship between cf-DNA and clinical manifestations should be performed.

## 5. Conclusion

In conclusion, our study using CLP mice revealed that the cf-DNA levels were elevated in the early phase of septic condition, implying a potential of cf-DNA to reflect the severity of sepsis and indicating its usefulness as a biomarker for the early detection of septic conditions. Unexpectedly, under septic conditions, it was moreover observed that cf-DNA was not derived from NETs produced by neutrophils, but mainly from host cells other than neutrophils. We hypothesize that the main source of cf-DNA might be dead tissue particles, such as necrotic cells. This may change the pathophysiological concept of cf-DNA formation during sepsis, at least in part, and further studies are needed to confirm this hypothesis.
